# Predicting OCT retinal ganglion cell volume from pattern ERGs and VEPs in children with suspected optic neuropathy in a tertiary referral setting

**DOI:** 10.1136/bmjophth-2024-001899

**Published:** 2025-03-23

**Authors:** Dorothy A Thompson, Katrina L Prise, Lisa Tucker, Dermot Roche, Richard Bowman, Benjamin E W Evans, Sian Handley, Oliver R Marmoy

**Affiliations:** 1 UCL Great Ormond Street Institute of Child Health, UCL, London, UK; 2 Clinical and Academic Department of Ophthalmology, Great Ormond Street Hospital for Children NHS Foundation Trust, London, UK; 3 Centre for Applied Research, School for Health Sciences, City University of London, London, UK

**Keywords:** Electrophysiology, Imaging, Optic Nerve

## Abstract

**Purpose:**

To determine the relationship between retinal ganglion cell (RGC) structure, measured by optical coherence tomography (OCT), and function measured by electrodiagnostic tests in children with suspected optic neuropathy.

**Methods:**

Children presenting consecutively with suspected optic neuropathy were investigated by visual electrophysiology. ISCEV Standard monocular pattern reversal visual evoked potential (PVEP) P100 and pattern electroretinogram (PERG) P50 and N95, amplitudes and peak times were collected, along with the N95 slope 30 ms after the P50 peak. OCT retinal nerve fibre layer (RNFL) thickness from each peripapillary sector and the average macular RGC volume in a 3.45 mm diameter circle were collated. The sensitivity (SENS) and specificity (SPEC) of abnormal visual electrophysiology measures in predicting OCT structural measures were estimated by receiver operating characteristic (ROC) area under the ROC curve (AUC).

**Results:**

Monocular PVEPs and PERGs from 30-degree stimulus fields and OCT RNFL and RGC volume were available from 42 children (84 eyes) aged 5.5–16.3 years (median 12.4 years). PVEP AUC was highest for predicting both RGC macular volume and temporal RNFL thinning (SENS 88%, SPEC 88%), followed by the PERG N95 slope (SENS 79%, SPEC 78%). PERG N95:P50 had the lowest SENS of 62% and SPEC of 61%, which were similar for all RNFL sectors.

**Conclusion:**

Abnormal PERG N95 slopes and PVEPs predicted severe loss of macular RGC volume and temporal sector RNFL with high diagnostic accuracy. These measures are important additions to the less specific PERG N95:P50, which was broadly sensitive to all RNFL sectors. The N95 slope and PVEPs are valuable objective functional markers of RGC health in children.

**WHAT IS ALREADY KNOWN ON THIS TOPIC**

- A low pattern electroretinogram (PERG) amplitude ratio (N95:P50) is used widely to identify retinal ganglion cell (RGC) disease and optic nerve dysfunction.

**WHAT THIS STUDY ADDS**

- The measurement of the PERG N95 slope or h30 (the N95 amplitude 30 ms after the P50 peak) markedly increases the specificity of PERGs in predicting optical coherence tomography (OCT) loss of macular RGC volume and temporal sector retinal nerve fibre layer thinning in children. Pattern reversal visual evoked potentials (PVEPs) have the highest sensitivity and specificity.

**HOW THIS STUDY MIGHT AFFECT RESEARCH, PRACTICE OR POLICY**

- The simple addition of the N95 slope measure, taken from the same clinical ISCEV Standard PERG waveform that yields the N95:P50 amplitude ratio, augments diagnostic specificity for predicting structural RGC loss. The PVEP offers a powerful indirect indicator of RGC health and structure in children unable to comply with OCT imaging.

## Introduction

 The differential diagnosis of paediatric optic neuropathy is challenging.^1^,^3^ Children can present with non-specific signs and symptoms, such as visual loss and headache, while the fundus examination may show anomalous, equivocally pale or possibly swollen optic nerve heads. Optical coherence tomography (OCT) provides measures of the optic nerve head structure, such as the thickness of the peripapillary retinal nerve fibre layer (RNFL), where ganglion cell axons enter the optic nerve head, and the volume of the macular retinal ganglion cell (RGC) layer comprising cell bodies and axons. Visual electrophysiology tests provide objective and complementary functional measures of RGC health and can localise the site of dysfunction along the visual pathway. The objectivity is valuable particularly for children who may be unable to describe their visual symptoms or give accurate subjective responses during behavioural neuro-ophthalmological assessments, such as a visual field test, or sustain fixation steadily enough for OCT image acquisition.

The pattern electroretinogram (PERG) and pattern reversal visual evoked potential (PVEP) are two clinical tests used commonly to investigate optic neuropathy.^4 5^ The main waveform components of the PERG, termed P50 and N95, and the PVEP, termed P100, are companion measures of RGC function along the visual pathway. Pharmacological block of spiking neurons in the retina by tetrodotoxin has been shown to completely abolish N95 and most of the P50 of the macaque PERG.^6^ Consequently, RGC activity is regarded to contribute 100% to the N95 and 75% to the P50. The P50 can become smaller, and earlier when RGC loss is severe, but even in cases of isolated RGC dysfunction, the P50 will not disappear entirely because it is driven by macular cones.^7^ The PVEP reflects the activation of the striate cortex by signals carried by RGC axons that form the optic nerve, decussate at the chiasm and synapse at the lateral geniculate nucleus before travelling the length of the visual pathway. At the striate cortex, the macular representation of the visual field is cortically magnified,^8^ and the PVEP reflects the macular RGC representation of the central 5–10 degrees of the visual field.^9^

The PERG P50 and PVEP P100 are interpreted together to exclude macular cone dysfunction as a cause of an abnormal PVEP. The PERG amplitude ratio (N95:P50) normalises P50 across individuals to reveal selective loss of N95 amplitude as an indicator of RGC dysfunction. A typical N95:P50 exceeds 1.1^10 11^ depending on the size of the stimulus field.^5^ This amplitude ratio has been used clinically for more than 30 years.^12^ More recently, Sustar Habjan *et al*^13^ proposed a new, additional PERG measure that quantifies the descending slope of the N95 over the 30 ms that follows the P50 peak.

The purpose of our study was to better understand the extent of structural RGC change which is predicted by abnormal electrophysiology measures. We compared PERG amplitude ratio, N95:P50, the N95 slope and PVEPs to OCT measures in children suspected of having optic neuropathy and/or RGC dysfunction. The electrophysiology measures served as the gold standard using predetermined and published reference limits from healthy subjects,^5 10 11^ within the framework of a Boolean outcome (normal vs abnormal/pass vs fail). We sought to determine the optimal cut-off values, which maximise sensitivity (SENS) and specificity (SPEC), for abnormal OCT structure using these different electrodiagnostic measures.

## Methods

We carried out a retrospective analysis of visual electrophysiological and OCT measures from children who presented consecutively to a tertiary referral ophthalmology unit for investigation of possible optic neuropathy over a 12-month period. Clinical notes were reviewed to extract age, sex, visual acuity (VA), clinical signs and symptoms and most recent diagnosis.

The visual electrophysiology tests followed international ISCEV Standards for PERG^14^ and PVEP^4^ using Espion E6 (Diagnosys, Massachusetts, USA) equipment. The PERGs were recorded with corneal fibre electrodes, positioned at the lower lid margin, referred to ipsilateral outer canthus electrodes. High-contrast 50’ check widths that phase reversed 3/s were presented in two field sizes, 30 and 15 degrees, each viewed at 1.2 m. Monocular PVEPs were measured from midline electrodes Oz and Iz, referred to a mid-frontal electrode, in response to ISCEV large (50’) and small (12’) check widths that phase reversed 3/s in the 30-degree field. The PERG is vulnerable to defocus and the PVEP to intrusion of slow wave electroencephalogram if a child becomes bored. To mitigate movement and fixation artefacts, the corneal reflection of the pattern was monitored by video camera, and data acquisition was paused whenever fixation was lost and resumed when the corneal reflection was regained. Attention was sustained by interspersing patterns with DVD cartoons while the soundtrack played continuously. In addition, the child was encouraged to report any changes in colour or shape of the fixation spot to help maintain fixation, focus and attention.

Children were included if they had measurements available from the same eye for PVEPs and PERGs from the larger 30-degree field and OCT measures of both RNFL and macular RGC volume at the same visit. Children were excluded if their PERG P50 amplitudes were below reference limits indicating maculopathy, or if flash electroretinography, which was carried out in all children, indicated retinal dystrophy.

The retinal structural measures were taken with the Heidelberg SPECTRALIS OCT (Heidelberg Engineering, Heidelberg, Germany) using Heyex2 acquisition software V.6.16.7.0 (hardware module HRA+OCT S3610-1FP), and the eye-tracking function enabled. Macular RGC volume scan (512 A-scans, 25 B-scans volume 20×20 degrees) centred around the fovea and a circular 12-degree peripapillary scan (1536 A-scans, with automatic real time mean (ART) ~23) centred around the optic nerve were included. Quality control used the consensus criteria for centration at the fovea and a signal strength exceeding 15 dB from "OSCAR IB" .^15^ This acronym identifies each of seven quality criteria by the first letter, [(O)=obvious problems including violation of the protocol; (S) poor signal strength defined as, 15 dB; (C) wrong centration of scan; (A) algorithm failure; (R) retinal pathology other than MS related; (I) illumination; and (B) beam placement]. All B-scans were auto-segmented, followed by visual inspection for segmentation accuracy and manual correction undertaken if required. Imaging and electrophysiology were acquired by different members of the team without knowledge of outcomes. It was not appropriate or possible to involve patients or the public in the design, or conduct, or reporting, or dissemination plans of our research.

### Analysis

**Figure 1:**
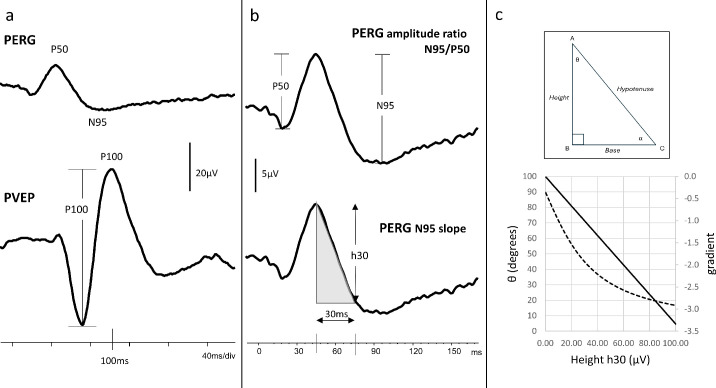
Electrophysiology waveforms and the measured components. Peak time and peak to trough amplitude of the pattern reversal visual evoked potential (PVEP) P100 and the pattern electroretinogram (PERG) N95 and P50 are measured as shown in panel (a). The PERG amplitude ratio, N95:P50, typically exceeds 1.1 (b). The N95 slope shaded in panel (b) is the linear gradient of the descending slope of N95 over the 30 ms that follows the P50 peak. The hypotenuse is proportional to height (h30) at 30 ms. Panel (c) shows the gradient has linear variation with height (h30), but that the angle tangent or sine is non-linear and is not helpful for comparison.

The midline PVEP P100 amplitude was measured from the preceding negative trough for both ISCEV check widths, large 50’ and small 12’ (figure 1a). The PERG amplitude ratio, N95:P50, was calculated for 50’ checks presented in 30 and 15-degree fields (figure 1b). The linear slope of the PERG N95 from the 30-degree field was calculated over 30 ms from the P50 peak. The slope gradient (h30/30) is proportional to h30 in all cases as the denominator is constant (figure 1c). Care was taken to use the gradient as a measure for comparison, rather than the angle, due to the non-linear relationship between the height of the triangle and the angle (see also Sustar Habjan *et al*.^13^

OCT measures of the macular RGC layer volume (containing RGC cell bodies) and RNFL thickness (containing the RGC axons) were extracted from the highest quality macular cube and RNFL scans available. The RGC layer volume scans were calculated using SPECTRALIS proprietary software after manually adjusting the circular templates to coincide with the fovea of the axial images. The averaged RGC volume from the 3.45 mm diameter circle template was used for all analyses. The summed RGC layer volumes within the Early Treatment Diabetic retinopathy Study (ETDRS) 6 mm diameter circle template were collected, but on inspection, the peripheral sectors of the template fell outside the extent of captured axial scans for children with non-central fixation. The RNFL inferior, superior, nasal, temporal sectors, sum of temporal sectors and global measures were collated. Examples of the OCT measures used are illustrated in online supplemental figure SF1.

A binary classification of function was used to define clinical PERG and PVEP measures as abnormal or normal compared with laboratory reference ranges. These acted as the gold standard, and OCT measures of structure acted as the test for receiver operating characteristic (ROC) curve calculations.

To assess the interocular agreement, the presence of each abnormal electrophysiology measure was cross-classified between the two eyes using the kappa (K) statistic measure of overall agreement corrected for chance^16^ in Excel. Other statistical analyses and the area under the ROC curve (AUC) were calculated using OriginPro 2022b (OriginLab, Northampton, Massachusetts, USA) for all structure measures, for all eyes together and for right eye (RE) and left eye (LE) independently. Non-parametric statistics were used to compare the logarithm of the minimum angle of resolution, LogMAR VA distributions for children grouped by abnormal and normal PVEPs, and the ranked comparators of RGC volumes from 3.45 and 6 mm diameter templates.

## Results

Complete datasets comprising 30-degree field PERGs, PVEPs and RNFL and RGC volume measures were available for 42 patients (84 eyes) aged 5.5–16.3 years (median 12.4 years). Four children did not comply with the 15-degree field PERG. These missing data were not associated with age, nor specific condition, and a separate analysis of PERG data from the smaller 15-degree field for each eye of 38 children was carried out.

The eventual diagnoses from available multidisciplinary team consensus were heterogenous and were grouped into four classifications: (1) hereditary optic atrophy (9 cases), (2) acquired optic atrophy, for example, from neuritis or glioma (8 cases), (3) swollen discs (13 cases) and (4) visual reduction with or without field loss or headaches (12 cases), providing a spectrum of disease. These are detailed in online supplemental table ST1 with subclassification of genetic or clinical diagnosis.

The number of eyes with abnormal visual electrophysiology measures compared with laboratory reference limits is tabulated in table 1.

**Table 1:** Cases of abnormal visual electrophysiology compared with reference limits

Test	Measure	Abnormal measure	Reference values	n
PERG	N95/P50 30° field	58% 49/84 eyes	Reference ratio >1.1	50
	N95/P50 15° field	45% 34/76 eyes	Reference ratio >1.3	50
	N95 slope 30° field	23% 18/84 eyes	Reference limit calculated slope −0.15or h30 < −4.5 μV	25
PVEP	P100 total	30% 25/84 eyes	Thompson *et al*^7^	697
By check width				
	P100 to 50’ only	7 eyes	PT <87 ms, >117 ms; amplitude <5 μV	
	P100 to 12’ only	4 eyes	PT <93 ms, >139 ms; amplitude <4 μV	
	P100 both 50’ and 12’	14 eyes	P100 abnormal to large and small check widths	

The number of eyes with abnormal electrophysiology measures is detailed with the reference limit used for each measurement. The number of subjects contributing to our unpublished PERG laboratory reference range is given in the right-hand column (n). PVEP reference data have been published.

PERGpattern electroretinogramPTpeak timePVEPpattern reversal visual evoked potential

Although only the averaged RGC volume from the 3.45 mm diameter circle template was used in the ROC analyses, there was a strong positive rank correlation with the interpolated RGC volume from the 6 mm diameter circle template 0.97 (Spearman correlation r(83)=0.93, p=2.8E-36), despite template clipping peripherally.

The median LogMAR VA differed for eyes with abnormal PVEPs compared with normal PVEPs, but there was considerable overlap in distribution; LogMAR 0.425 (IQR 0.08–0.85) compared with LogMAR 0.10 (IQR 0.0–0.40).

High-contrast LogMAR VA was weakly negatively correlated with the papillomacular temporal sector RNFL and macular RGC volume (Pearson correlation coefficient r(75)=−0.303, p=0.00788 and r(75)=−0.35, p=0.00186 at 0.05 level, respectively). These two structural OCT measures were strongly positively correlated with each other, r(83)=0.858, p=1.89E-25.

**Figure 2:**
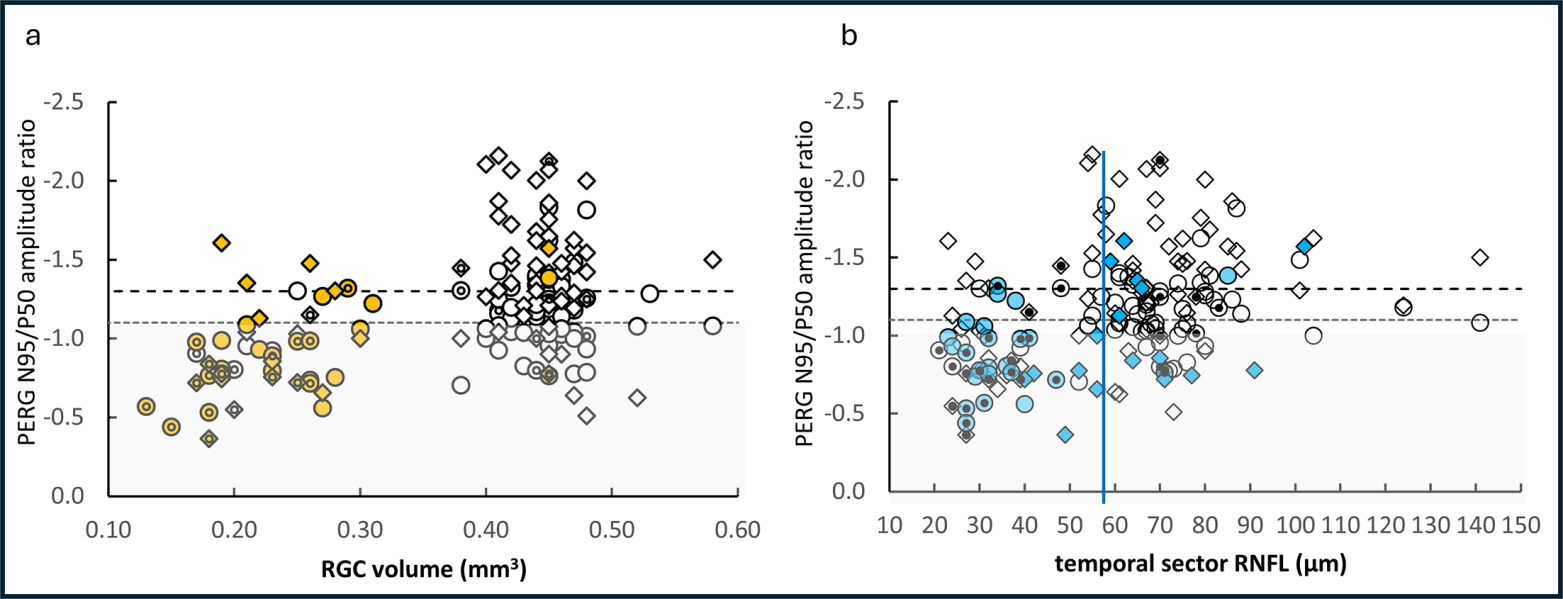
Scatter plot distribution of retinal ganglion cell (RGC) volume and retinal nerve fibre layer (RNFL) thickness against pattern electroretinogram (PERG) N95:P50. The amplitude ratio, N95:P50, for eyes from 30-degree (circle) and 15-degree (diamond) fields is plotted against the macular RGC layer volume from the 3.45 mm diameter template (a) and the temporal sector RNFL thickness (b). The lower reference limits for the ratios in each field size are shown by horizontal dotted lines. The closed symbols indicate abnormal pattern reversal visual evoked potentials (PVEPs). The small black dots indicate abnormal N95 slope. This distribution highlights the sensitivity of abnormal PVEPs to low RGC volume. The 5th centile estimated reference for temporal RNFL for children at 58 µm^19^ is shown as a vertical guideline in panel (b).

Scatter plots of the distribution of PERG amplitude ratio (N95:P50) versus RGC volume for each eye are shown in figure 2a and against RNFL thickness in figure 2b. Cases with abnormal PVEP and/or PERG N95 slope are overlaid as filled symbols. The reference limits defining abnormal amplitude ratios, N95:P50, are drawn as horizontal dotted lines such that points below the line are abnormal. The shaded area highlights the abnormal N95:P50 from the 30-degree field. It is noticeable that the distribution of abnormal PVEPs and abnormal N95 slope (filled symbols) clusters at the lower values for macular RGC layer volume and temporal RNFL thickness.

**Figure 3:**
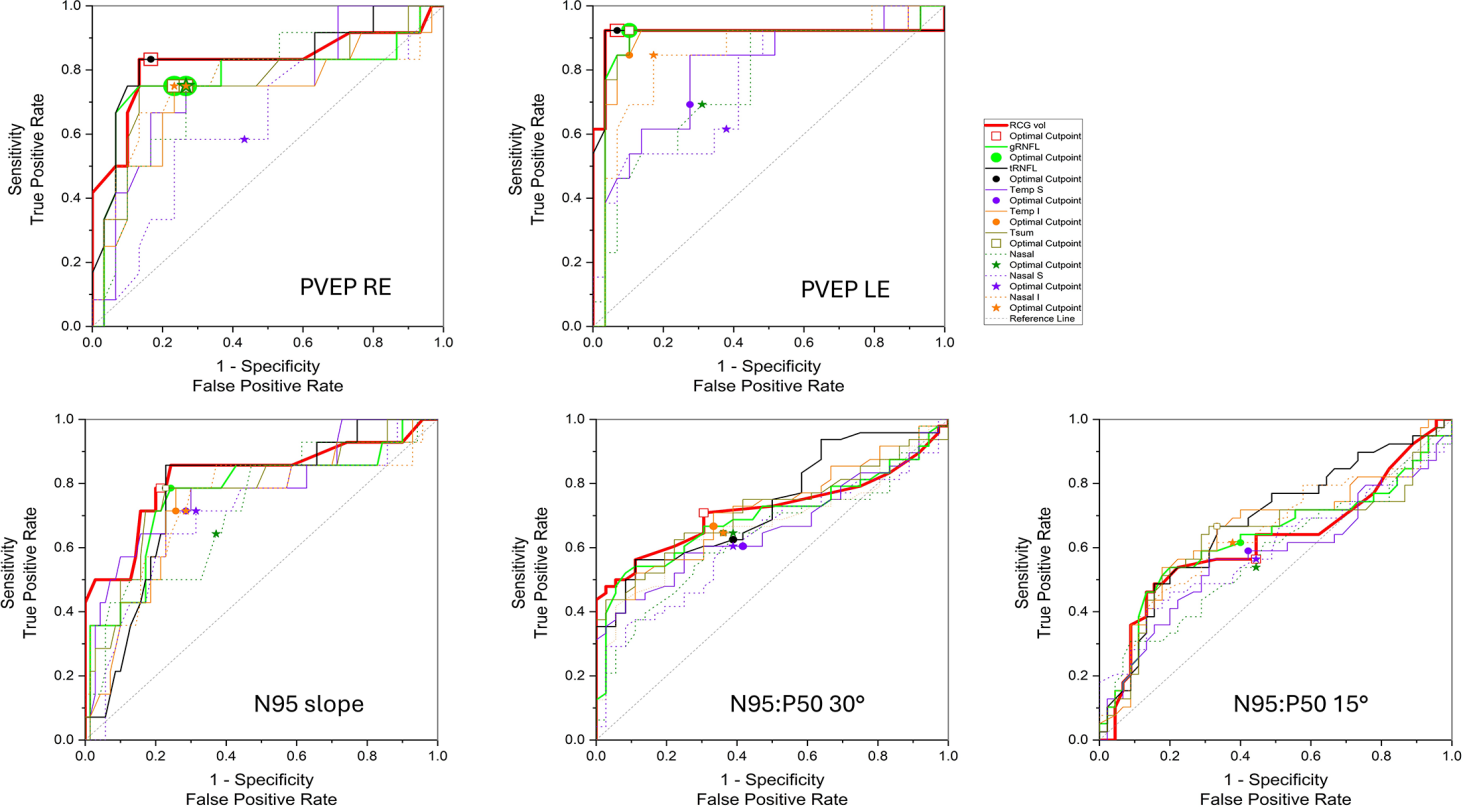
Receiver operating characteristic (ROC) curves for retinal nerve fibre layer (RNFL) thickness sectors and retinal ganglion cell (RGC) volume. ROC curves are plotted for the four different electrophysiological measures against the array of all optical coherence tomography (OCT) RNFL sector measurements and the macular RGC layer volume. Nasal sectors are dotted lines. These highlight the higher specificity of the pattern electroretinogram (PERG) N95 slope for thin temporal RNFL and low RGC volume compared with the PERG amplitude ratio, N95:P50. The pattern reversal visual evoked potentials (PVEPs) from either eye show the highest area under the ROC curve (AUC) for predicting abnormal OCT structure. RGC volume, macular retinal ganglion cell volume. LE, left eye; RE, right eye. RNFL prefix: g, global; n, nasal; ni, nasal inferior; ns, nasal superior; t, temporal; ti, temporal inferior; ts, temporal superior; tsum, total of temporal sectors t, ts and ti.

The cross-classification statistic kappa can range from +1 to −1 in which 0 is no agreement and 1 is perfect agreement. The agreement between the two eyes for each electrodiagnostic measure described by kappa ranged from 0.6 for PVEP to 0.135 for PERG amplitude ratio, N95:P50, in a 30-degree field. Hence, for the PVEP where the kappa was 0.6, the eyes were treated as being correlated as the agreement was high even by correcting by chance. For the other PERG measures, the kappa was low; N95 slope K=0.314, N95:P50 ratio in 30-degree field K=0.135 and PERG N95:P50 in the 15-degree field K=0.287, respectively, so the eyes were treated as being independent. ROC curves were calculated with electrophysiology measures as the reference gold standard and OCT measures as the diagnostic index test. Figure 3 shows the ROC analysis for each of these electrophysiological measures. (Online supplemental figure SF2 for completeness shows the ROC analysis with the PVEP from the RE and LE combined and for the other three PERG measures from each eye separately.)

Table 2 tabulates the cut-off balance between maximal SENS and SPEC with positive and negative predictive values of each electrophysiological measure for macular RGC volume and temporal RNFL sector thickness. (A data sheet documenting all OCT and corresponding electrophysiology outcome measures is provided with conditional formatting as online supplemental table ST2 with all corresponding ROC data for RE and LE, individually and combined, in online supplemental table ST3.)

**Table 2:** Summary table of AUC cut-off, SENS, SPEC, PPV and NPV

RGC macular volume					
	Cut-off	SEN	SPEC	PPV	NPV
PVEP all eyes	0.39	0.88	0.88	0.76	0.95
PVEP RE	0.39	0.83	0.83	0.67	0.93
PVEP LE	0.39	0.92	0.93	0.86	0.96
N95 slope all eyes	0.31	0.79	0.79	0.42	0.95
N95 slope RE	0.39	0.71	0.71	0.33	0.93
N95 slope LE	0.29	0.86	0.86	0.55	0.97
N95:P50 30° all eyes	0.44	0.71	0.69	0.76	0.64
N95:P50 30° RE	0.44	0.81	0.81	0.88	0.72
N95:P50 30° LE	0.40	0.59	0.90	0.87	0.67
N95:P50 15° all eyes	0.44	0.64	0.62	0.64	0.62
N95:P50 15° RE	0.43	0.48	0.53	0.61	0.40
N95:P50 15° LE	0.43	0.69	0.68	0.61	0.75

RNFL temporal sector					
	Cut-off	SEN	SPEC	PPV	NPV
PVEP all eyes	53.00	0.88	0.88	0.76	0.95
PVEP RE	53.00	0.83	0.83	0.67	0.93
PVEP LE	51.50	0.92	0.93	0.86	0.96
N95 slope all eyes	47.50	0.79	0.77	0.41	0.95
N95 slope RE	53.00	0.71	0.71	0.33	0.93
N95 slope LE	44.50	0.86	0.80	0.46	0.97
N95:P50 30° all eyes	64.50	0.63	0.61	0.68	0.55
N95:P50 30° RE	64.00	0.65	0.69	0.77	0.55
N95:P50 30° LE	64.50	0.59	0.55	0.59	0.55
N95:P50 15° all eyes	64.50	0.67	0.65	0.67	0.65
N95:P50 15° RE	64.00	0.65	0.73	0.79	0.58
N95:P50 15° LE	63.50	0.69	0.73	0.65	0.76

The cut-off balance between maximal sensitivity (SENS) and specificity (SPEC) tabulated with positive and negative predictive values is tabulated for the macular RGC volume and temporal RNFL sector for each electrophysiological measure.

AUCarea under the ROC curveLEleft eyeNPVnegative predictive valuePPVpositive predictive valuePVEPpattern reversal visual evoked potentialREright eyeRGCretinal ganglion cellRNFLretinal nerve fibre layer

## Discussion

We evaluated how standard clinical electrophysiological measures of RGC function predicted OCT structural measures of RGC volume and RNFL in a cohort of children investigated for suspected optic neuropathy. Our analyses revealed important and intriguing distinctions in the SPEC and SENS of different electrophysiological measures. The PVEP P100 discriminated abnormal OCT measures with high SENS and SPEC (AUC 0.87), particularly the macular RGC volume. Next in rank with a good AUC of 0.82 was the PERG N95 slope taken 30 ms after P50, a new N95 measure, which showed a high SPEC for identifying temporal sector RNFL thinning compared with the more commonly used PERG amplitude ratio, N95:P50, which had the lowest AUC of 0.72.

The addition of the new N95 slope measurement enhanced the SPEC of the PERG for predicting thinning of the RNFL temporal sector and low macular RGC volume. One practical advantage is the easier identification and measurement of the N95 slope compared with N95 peak to trough amplitude. The endpoints of the slope start at the P50 peak and end 30 ms later and can be identified with more certainty than the minima of the broad N95 trough. A second advantage is that the N95 slope gradient is proportional to the amplitude drop h30 (figure 1b) (run and rise) and can be cursored directly from the waveform. In this cohort, the slope derived from h30 corresponds with the published slope values calculated from offline linear regression.^13^ The h30 measure offers an efficient clinical alternative to the extra steps involved in the data export for linear regression analysis.^13^

It is interesting to consider why the initial 30 ms of the N95 slope should better reflect thinning of the temporal RNFL sector than the amplitude ratio, N95:P50, which shows less but more generalised SENS to thinning of all RNFL sectors (figure 3, table 2).

One hypothesis may involve the different RGC origins of positive and negative PERG components. An elegant analysis of retinal conduction times of multifocal PERGs suggests RGC cell bodies account for positive components (P50), and RGC axons the negative components (N95).^17^ The ratio of PERG amplitudes, N95:P50, identifies relative predominance of N95 loss. As RGC axons account for the N95, this may explain the broad SENS of the N95:P50 ratio to thinning across all RNFL sectors.

In contrast, the N95 slope is proportional to h30, which is an absolute amplitude influenced by the preceding P50 amplitude. The h30, N95 slope, could signify dysfunction of both RGC cell bodies and axons and therefore more severe disease. Indeed, in our study, a shallow N95 slope predicted more severe structural OCT thinning, in particular, of the temporal RNFL sector representing the papillomacular bundle.

Visual electrophysiology abnormalities were defined as measures falling outside the clinical reference limits. The PERG and PVEPs were recorded to ISCEV Standards^4 14^ and the reference ranges used for the analysis agree with other published reference data for healthy subjects; PERG N95:P50 and N95 slope^10 11 18^ and PVEP P100.^7^ Our findings may be translated readily, therefore, to other visual electrophysiology clinics that use ISCEV Standard tests.

In this paediatric cohort, we found PERG ratio measures were abnormal more often than PVEPs and PERG slope. The PERG ratios were abnormal in some eyes when structural OCT measures were within the reference ranges published for children.^19 20^ Such discrepancies between structure and function support the supposition that a reduced PERG N95 to P50 amplitude ratio reflects early RGC axonal dysfunction prior to structural change. Indeed, a number of PERG and perimetric studies in glaucoma show functional RGC change precedes structural change.^21 22^ Future longitudinal studies are needed to elaborate the similar time course for children.

We used a 3.45 mm diameter circular OCT template centred over the fovea to assess RGC layer volume, because in some children the peripheral sectors of the larger 6 mm diameter ETDRS template were clipped by non-centred 20-degree scans due to off-centre fixation or by shorter scan sizes associated with some axial lengths. Al-Hawasi and Lagali estimated the range of RGC volumes in the ETDRS 6 mm template as 0.97–1.33 mm^3^ using the Heidelberg SPECTRALIS OCT system 30×20 degree Retina DENS programme centred on the macula.^23^ This agreed with RGC volume data from 6 mm-centred templates in this study, taken from children with no electrophysiological or RNFL abnormality. We also found a strong ranked correlation between the interpolated RGC volume in 6 mm and the 3.45 mm diameter templates. For future studies, an OCT scan size for RGC volume assessment perhaps needs to be standardised to ensure the inclusion of children or patients with poor central vision or eccentric fixation. Recentring of macular scans has been reported to be necessary in up to 25% of control scans.^23^ The 3.45 mm diameter template equates approximately to 11.5 degrees of central retina. It is estimated that 32% of the total number of RGCs is in the central 10 degrees,^24^ increasing to 50% in the central 16 degrees around the fovea.^25^ Although we may estimate the mean RGC receptive field count associated with OCT and PERG stimulus fields from recent work,^24 26^ the high interindividual variability currently precludes an individual analysis.^24^

Abnormal PVEPs tended to be associated with severe structural change, which was most common in group 1, who had hereditary or suspected hereditary optic atrophy because of reported family history, or group 2, who had episodes of optic neuritis. This is highlighted by the conditional formatting in online supplemental table ST2. Only two PVEP P100 measures were used in the analysis; the amplitude and peak time of the midline P100 of two check widths. In clinical paediatric practice, PVEP SENS is improved further by including other check widths, analysing the shape and definition of the waveform and the transoccipital distribution from lateral electrodes.

In some cases, discrepancies in PVEP and PERG can be related to their dependence on the function of different regions of the central retina and different RGC population densities. The PERG is summed from activity over a wide 30-degree or 15-degree square area. The PVEP depends on a smaller foveal field. This is illustrated in online supplemental figure 1b which highlights an outlier of normal PVEP yet low RGC volume with abnormal summed PERG. This is explained by the distribution of RNFL thinning in areas temporal to the fovea and sparing the papillomacular bundle. Other outliers include abnormal PVEPs in cases of full macular RGC volume. The PVEP identifies functional loss along the visual pathway to the striate cortex. A normal PERG with abnormal PVEP may reflect dysfunction that affects the passage of RGC signals along the visual pathway before retrograde structural degeneration occurs. Interestingly, there were differences in PVEPs to the two check widths. In this cohort, the P100 to small checks was vulnerable to primary RGC disease and optic atrophy (group 1), while the P100 to large checks was vulnerable to inflammatory processes (group 2). This confirms the importance of including different check widths in the PVEP recording. The use of a range of check widths has been shown to be more sensitive, particularly in early optic nerve disease.^27^ The high SENS and SPEC of the PVEP support its use to investigate children unable to comply with PERG recording when optic neuropathy is suspected.

High-contrast VA is used widely as a gold standard measure of treatment outcome in optic neuropathy, such as defining clinically relevant benefit in Leber Hereditary Optic Neuropathy, LHON.^1^ In cases of optic atrophy, high-contrast VA can be widely discrepant and typically better than PVEP estimates of visual function.^28^ This may be explained by examining the relationship of PVEP amplitude with contrast. The P100 amplitude saturates at ~20% contrast and any loss of P100 amplitude can be translated to a loss of stimulus contrast. The PVEP may be considered to reflect a patient’s spatial vision at low contrast, a more common real-world scenario. Although children with optic neuropathy may have reasonable high-contrast VA such as the child in online supplemental figure 1a, who had the same high-contrast LogMAR VA 0.10 in affected and fellow unaffected eyes, their visual impairment will become apparent and exacerbated in low-contrast conditions.

This study was not immune to the limitations commonly introduced in observational retrospective studies, particularly clinical studies involving children. A primary limitation arose due to missing elements of data, impacting both the cohort size and age range. Mostly, this was limited by the absence of the high-quality OCT structural measures used in this study. Volume scans are not possible to obtain in all children; some children are unable to comply with chin rests, or fixate steadily for long enough periods without blinking or movement. In some circumstances, swollen optic discs confounded the OCT measures, and the RGC and RNFL layers could not be segmented reliably due to ambiguous retinal lamination. OCT imaging is less amenable to the interrupted averaging which may be used to record PERG and PVEPs successfully in distractable, restless children.

This study determined the relative predictive power of different electrophysiological measures, including the PERG N95 to P50 amplitude ratio, the N95 slope and PVEP, on structural RGC loss when optic neuropathy is suspected at presentation. Optic neuropathy can be bilateral, unilateral or asymmetric. To account for the intereye correlation before the AUC calculation, the kappa statistic was used to assess the level of interocular agreement corrected for change agreement.^16^ Table 2 shows similar AUC and cut-off for the PVEP when the data were analysed by individual eye, or when RE and LE were combined. This reflects the high SPEC and SENS of PVEPs to RGC and RNFL loss.

The heterogeneity of disease and diagnosis in the cohort strengthened the analysis by providing a wide spectrum of disease that helps delineate the structure versus function association at the preliminary investigation. Although an abnormal PVEP discriminated children with optic atrophy in groups 1 and 2, more data are required to ascribe characteristics to specific conditions. It is not unusual for the final diagnosis to be unknown in some children; a 5-year study of 143 children under 16 years with optic atrophy identified the cause in only 53%, after excluding those with inherited retinal dystrophies. All children in our cohort had normal flash ERGs excluding retinal dystrophy as a cause of optic atrophy.^29^ The OCT measurement for children in group 3 with suspicious disc swelling highlighted the challenge of using imaging to detect incipient nerve fibre loss. Group 4, comprising symptomatic children, in contrast, showed a greater proportion of borderline OCT measures, exemplifying the challenge of managing these children. In this group, 3/12 were diagnosed eventually with functional non-organic visual loss (online supplementaltable ST2).

The main strength of this study is its representation and applicability to the real-world clinical investigation of children in whom optic neuropathy is suspected. The data are pertinent for, and can be translated to, general clinical practice using ISCEV Standard electrophysiology and reference ranges that can cross centres with appropriate validation.^30^

This is the first study, to our knowledge, to apply the new PERG N95 slope analysis for children with optic neuropathy. We showed that abnormal PVEPs and abnormal N95 slopes predict those children most likely to have suffered RGC structural loss. This is an important outcome that informs the interpretation of visual electrophysiology in young children for whom high-quality OCT is not possible.

## Conclusion

Combining the PERG N95 slope analysis with the more common PERG amplitude ratio, N95:P50, enhances PERG discrimination of abnormal RGC structure, specifically temporal RNFL and macular RGC layer volume. The PVEP, produced to two check widths, combined with PERG measures, provides a functional RGC profile which can predict OCT RGC structure in children with suspected optic neuropathy.

## supplementary material

10.1136/bmjophth-2024-001899 online supplemental file 1

10.1136/bmjophth-2024-001899 online supplemental file 2

## Data Availability

All data relevant to the study are included in the article or uploaded as supplementary information.
