# Supplementary material for: Predicting OCT retinal ganglion cell volume from pattern ERGs and VEPs in children with suspected optic neuropathy in a tertiary referral setting
Source: BMJ Open Ophthalmol. 2025 Mar 23;10(1):e001899. doi: 10.1136/bmjophth-2024-001899 (PMC11931969; doi:10.1136/bmjophth-2024-001899)
Supplement: online supplemental file 1 [file bmjophth-10-1-s001.pdf]

Supplementary Figure SF1.

OCT measures in two cases

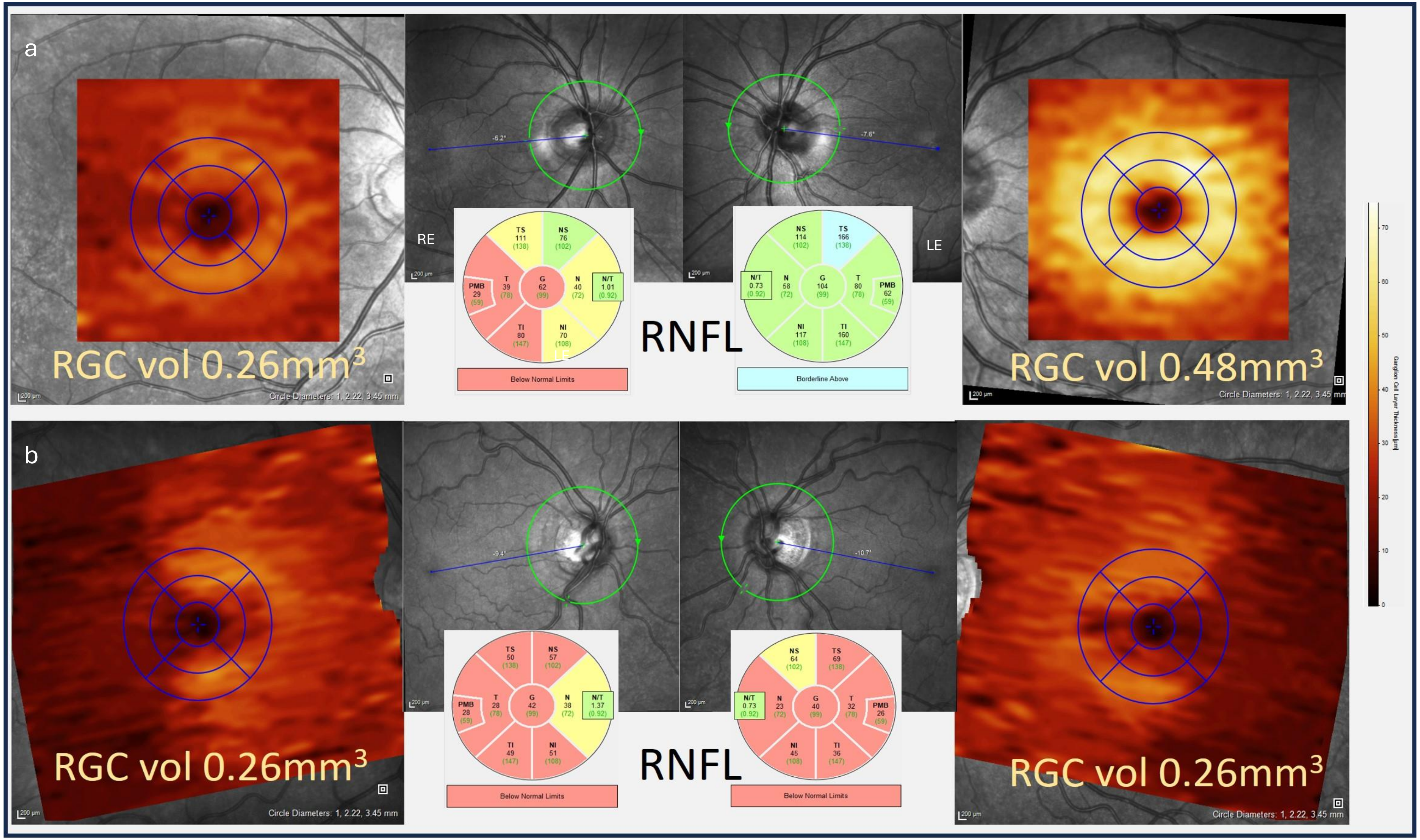

Supplementary Figure SF1 Legend.

OCT measures in two cases

Top row, Fig 1a. The OCT measures are shown for a 6y boy, 3 months after an episode of right eye MOG-ab optic neuritis associated with Myelin Oligodendrocyte Glycoprotein Antibody disease (MOGAD).

This highlights the striking inter-ocular difference in macular RGC volume and RNFL. The macular RGC layer volume scans are superimposed over the infra-red fundus images for RE and LE and show the 3.45mm template centred on the fovea. The Left eye macular RGC volume is seen as the typical yellow halo around the fovea, indicating healthy RGC macular volume of 0.48 mm^3. The RE macular RGC volume is 0.26mm^3, (~ 50% that of the LE), showing post neuritis loss of RE RGC. In the centre panel are the RFNL thickness maps for each eye. The sectors coloured red (RE temporal and temporal inferior) are thinner than the reference data, yellow sectors are borderline and green within the reference range. **LogMAR VA was the same RE=LE =0.1**. The RE PrVEP to small 12' check widths was abnormal and the RE 15-degree field PERG N95:50 was abnormal 0.87. The LE PVEP and PERG were within reference range.

Bottom row Fig 1b. The OCT measures for a 16y girl, who has optic atrophy and an older brother with optic atrophy. No relevant variants were found on whole genome sequencing, followed by optic neuropathy panels, mitochondrial disorders, and cerebral malformations.

The posterior pole OCTs show bilaterally low macular RGC volumes ~0.26mm^3, associated with striking bi-temporal, hemi macular retina RGC volume loss and relative sparing of the papillo-macular regions, despite diffuse, multisector RNFL thinning. **LogMAR VA was the same RE=LE =0.0.** The PVEP was within reference range, but the 15-degree field PERG N95:P50 was abnormal 0.72.

Supplemental Figure SF2.

ROC analysis for PVEP, (RE and LE), and monocular PERG measures for all OCT measures

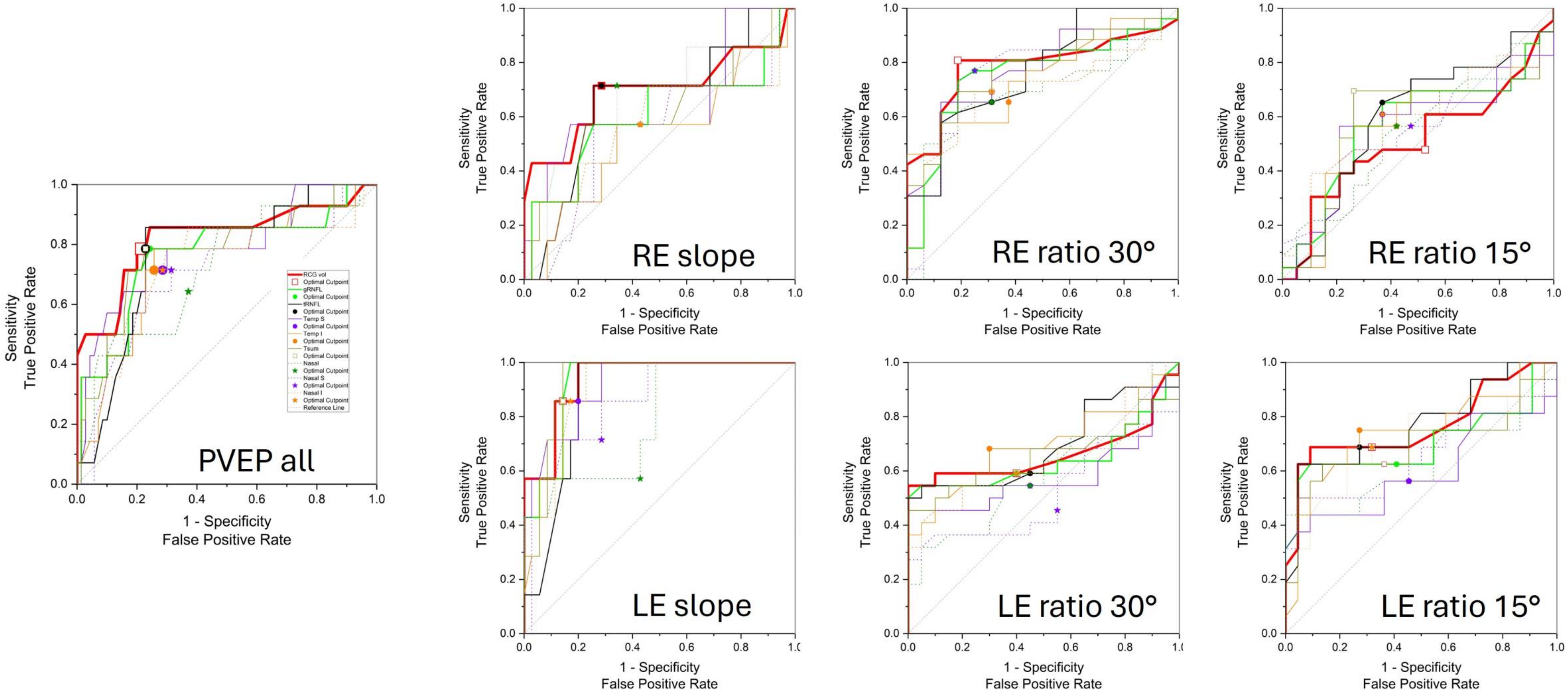

Supplemental Figure SF2 Legend.

ROC analysis for PVEP, (RE and LE), and monocular PERG measures for all OCT measures

ROC curves are plotted for the four different electrophysiology measures for the PVEP with both eyes combined and for the other three PERG measures with each eye separately, against the array of all OCT RNFL sector measurements and the macular RGC layer volume. This complements Figure 4. Nasal sectors are dotted lines. These highlights the higher specificity of the PERG N95 slope for thin temporal RNFL and low RGC volume compared to the PERG amplitude ratio, N95:P50. The PVEPs from show the highest AUC for predicting abnormal OCT structure.

Abbreviations: RGC vol macular retinal ganglion cell volume. RNFL prefix: g global, t temporal, ts temporal superior, ti temporal inferior, tsum total of all temporal sectors (t, ts and ti), ns nasal superior, n nasal, ni nasal inferior.
