# Supplementary material for: Predicting OCT retinal ganglion cell volume from pattern ERGs and VEPs in children with suspected optic neuropathy in a tertiary referral setting
Source: BMJ Open Ophthalmol. 2025 Mar 23;10(1):e001899. doi: 10.1136/bmjophth-2024-001899 (PMC11931969; doi:10.1136/bmjophth-2024-001899)
Supplement: online supplemental file 2 [file bmjophth-10-1-s002.pdf]

SUPPLEMENTARY TABLES pages 1-5

SUPPLEMENTARY TABLE ST1. **Confirmed or working diagnosis of children presenting with suspected optic neuropathy.** Abbreviations: *OPA1* optic atrophy type 1 gene; OA optic atrophy; FH family history; CRION chronic relapsing inflammatory optic neuritis; MOGAD myelin oligodendrocyte glycoprotein antibody disease; RRMS relapsing remitting multiple sclerosis; IIH Idiopathic intracranial hypertension; NPL no perception of light.

Group	Diagnosis		Number children (n)	Age range (months)
**1**	**Hereditary optic atrophy**	genetically confirmed *OPA1* variant	3	66-131
		OA positive FH not genetically identified	6	92-196
**2**	**Acquired optic atrophy**	Optic neuritis, CRION, MOGAD, RRMS	5	78-178
		Optic pathway glioma	1	130
		Unknown	2	
**3**	**Disc swelling**	IIH	6	105-182
		Optic disc drusen confirmed by ultrasound	2	127-154
		Craniosynostosis/ARFID/Unknown	3	178
		Chronic renal disease	1	117
		Bechet’s panuveitis	1	176
**4**	**Visual complaints and/or headaches**	Unexplained reduced VA (one or both eyes) or visual field loss or visual disturbance or cognitive change (LogMAR VA 0.34 to 1.00)	6	72-181
		Headaches (LogMAR VA -0.01 to 0.26)	3	138-193
		Functional or non-organic vision loss proven clinically (LogMAR VA 0.3 to NPL worse eye)	3	112-191

SUPPLEMENTARY TABLE ST2. **Data table with conditional formatting.** All data are presented for each subject. OCT values below the reference data shaded red, amber are borderline. Electrodiagnostic (EDT) data red, abnormal =1 or green, within reference range.

	OCT measure									EDT (1=abnormal)					
	RGC	RNFL sector								PVEP	PERG				
diagnostic group 1-4	vol (mm^3)	Global	Temp	Temp Sup	Temp Inf	Temp Sum	Nasal	Nasal Sup	Nasal Inf	P100 50'&12'	N95 slope	N95 30deg	N95 15deg	EYE	subject
1	0.19	62	23	90	59	172	55	111	78	1	0	1	0	RE	1
1	0.22	61	24	93	66	183	51	111	71	1	0	1	1	LE	1
1	0.23	70	32	110	80	222	58	116	73	1	0	1	1	RE	2
1	0.21	65	26	97	77	200	62	80	90	1	0	1	1	LE	2
1	0.2	70	24	121	80	225	72	95	73	1	0	1	1	RE	3
1	0.17	73	21	118	76	215	72	120	86	1	1	1	1	LE	3
1	0.23	78	39	150	100	289	56	108	75	0	0	1	1	RE	4
1	0.19	77	36	140	99	275	61	115	70	1	0	1	1	LE	4
1	0.23	42	27	50	50	127	37	56	49	0	0	1	1	RE	5
1	0.25	40	32	68	35	135	23	63	44	0	1	1	1	LE	5
1	0.19	52	30	73	75	178	41	80	48	1	1	1	1	RE	6
1	0.18	49	27	71	72	170	43	58	52	1	1	1	1	LE	6
1	0.29	73	34	104	94	232	62	111	84	1	1	0	-	RE	7
1	0.31	83	38	120	86	244	73	126	107	1	0	0	-	LE	7
1	0.25	73	30	115	72	217	67	116	84	0	0	1	1	RE	8
1	0.21	65	27	109	59	195	55	118	67	1	0	1	0	LE	8
1	0.38	77	52	102	93	247	75	89	77	0	0	1	1	RE	15
1	0.38	77	48	112	97	257	66	104	75	0	1	0	0	LE	15
2	0.15	45	27	38	68	133	39	74	51	1	1	1	-	RE	9
2	0.13	53	31	88	91	210	29	75	53	1	1	1	-	LE	9
2	0.27	62	40	113	81	234	39	74	72	0	0	1	1	RE	10
2	0.48	104	80	166	161	407	57	113	118	0	0	0	0	LE	10
2	0.42	116	67	137	144	348	83	147	201	0	0	0	0	RE	11
2	0.26	71	41	95	98	234	72	72	76	1	1	1	1	LE	11
2	0.17	71	39	92	103	234	62	90	77	1	1	1	1	RE	12
2	0.18	64	37	101	59	197	53	91	83	1	1	1	1	LE	12
2	0.27	56	34	111	95	240	34	50	56	1	0	0	1	RE	14
2	0.3	56	31	121	110	262	20	45	66	1	0	1	1	LE	14
2	0.26	59	29	95	66	190	53	75	69	1	0	1	0	RE	16
2	0.28	66	32	103	83	218	53	90	79	1	0	1	1	LE	16
2	0.45	91	71	153	158	382	44	93	93	1	1	1	1	RE	18
2	0.45	102	85	149	199	433	48	91	112	0	0	0	1	LE	18
2	0.4	74	54	122	115	291	44	74	86	0	0	1	0	RE	41
2	0.41	78	55	116	111	282	54	92	92	0	0	0	0	LE	41
3	0.26	144	47	142	146	335	160	187	267	1	1	1	-	RE	17
3	0.41	183	83	223	230	536	163	233	285	0	0	0	-	LE	17
3	0.47	207	60	192	177	429	215	402	335	0	0	0	1	RE	19
3	0.46	163	63	260	154	477	164	212	221	0	0	0	1	LE	19
3	0.48	127	78	158	214	450	80	169	206	1	1	1	1	RE	20
3	0.47	115	72	190	160	422	62	175	130	0	0	1	0	LE	20
3	0.43	214	76	217	172	465	283	268	336	0	0	1	1	RE	21
3	0.45	138	86	158	244	488	90	167	182	0	0	0	0	LE	21
3	0.45	123	80	189	178	447	95	160	107	0	0	0	1	RE	22
3	0.4	142	74	217	160	451	112	245	139	0	0	1	1	LE	22
3	0.43	88	68	104	139	311	66	109	80	0	0	1	1	RE	23
3	0.44	91	64	101	137	302	63	123	109	0	0	1	0	LE	23
3	0.42	101	76	174	165	415	60	107	89	0	0	1	0	RE	24
3	0.45	106	64	123	166	353	78	149	127	0	0	0	0	LE	24
3	0.47	112	124	161	158	443	61	116	91	0	0	0	1	RE	25
3	0.46	104	88	136	155	379	81	100	106	0	0	0	0	LE	25
3	0.47	106	75	136	157	368	89	106	119	0	0	1	0	RE	27
3	0.58	169	141	195	215	551	137	239	150	1	0	1	0	LE	27
3	0.47	137	101	223	221	545	82	183	105	0	0	0	1	RE	32
3	0.47	183	104	240	301	645	104	246	259	0	0	1	0	LE	32
3	0.48	132	87	181	192	460	102	154	153	0	0	0	0	RE	35
3	0.48	127	80	189	170	439	97	161	138	0	0	1	0	LE	35
3	0.46	73	54	68	75	197	97	48	93	0	0	1	1	RE	37
3	0.48	164	73	303	194	570	103	274	192	0	0	1	1	LE	37
3	0.45	104	82	139	164	385	73	116	102	0	0	0	-	RE	39
3	0.44	109	79	138	165	382	86	144	96	0	0	0	-	LE	39
4	0.41	87	67	119	129	315	59	80	114	0	0	1	1	RE	13
4	0.42	87	64	115	149	328	60	81	105	0	0	0	0	LE	13
4	0.42	96	69	132	134	335	82	109	94	0	0	1	0	RE	26
4	0.41	85	67	107	150	324	62	92	84	0	0	0	0	LE	26
4	0.45	97	61	128	118	307	76	143	114	0	0	0	1	RE	28
4	0.45	94	58	102	110	270	71	157	130	0	0	0	0	LE	28
4	0.41	90	69	125	141	335	56	101	99	0	0	1	0	RE	29
4	0.42	86	55	123	148	326	55	94	100	0	0	0	0	LE	29
4	0.46	105	74	164	123	361	81	127	113	0	0	0	0	RE	30
4	0.46	101	66	147	132	345	74	133	113	0	0	1	1	LE	30
4	0.43	92	60	105	92	257	96	101	127	0	0	1	1	RE	31
4	0.41	91	57	115	108	280	83	107	116	0	0	0	0	LE	31
4	0.45	89	70	136	121	327	72	88	80	0	0	0	0	RE	33
4	0.44	91	70	131	133	334	70	99	85	0	0	0	0	LE	33
4	0.44	105	81	131	146	358	76	120	129	0	0	0	0	RE	34
4	0.45	108	70	159	150	379	75	134	133	0	0	1	0	LE	34
4	0.44	101	61	144	122	327	86	128	120	0	0	0	0	RE	36
4	0.44	100	65	147	140	352	74	112	124	0	0	0	0	LE	36
4	0.44	96	75	127	140	342	75	105	96	0	0	0	0	RE	38
4	0.45	95	79	129	144	352	59	124	83	0	0	0	0	LE	38
4	0.44	111	67	159	148	374	107	119	113	0	0	0	0	RE	40
4	0.45	108	67	144	134	345	100	148	104	0	0	1	1	LE	40
4	0.52	119	61	135	129	325	116	149	184	0	0	1	1	RE	42
4	0.53	119	70	129	161	360	106	157	157	0	0	0	0	LE	42

SUPPLEMENTARY TABLE ST3. **All data analysis results** tabulated for AUC cut off balance between sensitivity and specificity with positive and negative predictive values, arranged all data right and left eye combined, right eye and left eye separately.

**ALL DATA**																	
**PVEP all**	State									1= positive for condition ie abnormal, 0=normal							
positive 1	25																
negative 0	59									1	0	1	0	a/(a+c)	d/(b+d)	a/(a+b)	d/(c+d)
	**Area**	**SE**	**Asymptotic F**	**95% LCL**	**95% UCL**	**cut off**	**1-SPEC**	**SEN**	**SPEC**	**a**	**b**	**c**	**d**	**Sens**	**Spfc**	**PPV**	**NPV**
RCG vol	0.8725	0.04068	7.62E-08	0.79281	0.95227	0.38983	0.11864	0.88	0.88136	22	7	3	52	0.8800	0.8814	0.7586	0.9455
gRNFL	0.8356	0.05078	1.28E-06	0.73607	0.93512	84	0.16949	0.84	0.83051	21	10	4	49	0.8400	0.8305	0.6774	0.9245
tRNFL	0.8776	0.04126	5.06E-08	0.79675	0.9585	53	0.11864	0.88	0.88136	22	7	3	52	0.8800	0.8814	0.7586	0.9455
Temp S	0.7739	0.0564	7.74E-05	0.66337	0.88443	120.5	0.27119	0.72	0.72881	18	16	7	43	0.7200	0.7288	0.5294	0.8600
Temp I	0.8112	0.05375	7.10E-06	0.70584	0.91653	110.5	0.18644	0.84	0.81356	21	11	4	48	0.8400	0.8136	0.6563	0.9231
Tsum	0.8312	0.0509	1.76E-06	0.73143	0.93094	277.5	0.15254	0.84	0.84746	21	9	4	50	0.8400	0.8475	0.7000	0.9259
Nasal	0.7576	0.05799	2.01E-04	0.64397	0.87129	62.5	0.28814	0.72	0.71186	18	17	7	42	0.7200	0.7119	0.5143	0.8571
Nasal S	0.6844	0.06295	0.00779	0.56103	0.80778	110	0.42373	0.56	0.57627	14	25	11	34	0.5600	0.5763	0.3590	0.7556
Nasal I	0.8003	0.05278	1.46E-05	0.6969	0.90378	89.5	0.23729	0.76	0.76271	19	14	6	45	0.7600	0.7627	0.5758	0.8824
**PERG h30 slope**	State																
positive	14																
negative	70									1	0	1	0	a/(a+c)	d/(b+d)	a/(a+b)	d/(c+d)
	**Area**	**SE**	**Asymptotic F**	**95% LCL**	**95% UCL**	**cut off**	**1-SPEC**	**SEN**	**SPEC**	**a**	**b**	**c**	**d**	**Sens**	**Spfc**	**PPV**	**NPV**
RCG vol	0.823	0.05156	1.45E-04	0.7219	0.92402	0.305	0.21429	0.78571	0.78571	11	15	3	55	0.7857	0.7857	0.4231	0.9483
gRNFL	0.7684	0.06402	0.0016	0.64289	0.89384	77.5	0.24286	0.78571	0.75714	11	17	3	53	0.7857	0.7571	0.3929	0.9464
tRNFL	0.7725	0.0783	0.00135	0.61898	0.92592	47.5	0.22857	0.78571	0.77143	11	16	3	54	0.7857	0.7714	0.4074	0.9474
Temp S	0.7821	0.06202	9.04E-04	0.66058	0.9037	115.5	0.28571	0.71429	0.71429	10	20	4	50	0.7143	0.7143	0.3333	0.9259
Temp I	0.7357	0.07303	0.00556	0.59258	0.87884	101.5	0.25714	0.71429	0.74286	10	18	4	52	0.7143	0.7429	0.3571	0.9286
Tsum	0.776	0.06547	0.00117	0.6477	0.90434	259.5	0.22857	0.78571	0.77143	11	16	3	54	0.7857	0.7714	0.4074	0.9474
Nasal	0.7189	0.07308	0.01004	0.57565	0.86211	62.5	0.37143	0.64286	0.62857	9	26	5	44	0.6429	0.6286	0.2571	0.8980
Nasal S	0.7061	0.07551	0.01533	0.55812	0.85412	104.5	0.31429	0.71429	0.68571	10	22	4	48	0.7143	0.6857	0.3125	0.9231
Nasal I	0.7464	0.06509	0.00375	0.61885	0.874	85.5	0.28571	0.71429	0.71429	10	20	4	50	0.7143	0.7143	0.3333	0.9259
**Ratio 30deg**	State																
positive	48																
negative	36									1	0	1	0	a/(a+c)	d/(b+d)	a/(a+b)	d/(c+d)
	**Area**	**SE**	**Asymptotic F**	**95% LCL**	**95% UCL**	**cut off**	**1-SPEC**	**SEN**	**SPEC**	**a**	**b**	**c**	**d**	**Sens**	**Spfc**	**PPV**	**NPV**
RCG vol	0.719	0.06319	6.24E-04	0.59519	0.84288	0.435	0.30556	0.70833	0.69444	34	11	14	25	0.7083	0.6944	0.7556	0.6410
gRNFL	0.6985	0.06333	0.00193	0.57438	0.82261	94.5	0.33333	0.66667	0.66667	32	12	16	24	0.6667	0.6667	0.7273	0.6000
tRNFL	0.7355	0.0566	2.34E-04	0.62461	0.84646	64.5	0.38889	0.625	0.61111	30	14	18	22	0.6250	0.6111	0.6818	0.5500
Temp S	0.6623	0.0642	0.01123	0.53649	0.78816	130	0.41667	0.60417	0.58333	29	15	19	21	0.6042	0.5833	0.6591	0.5250
Temp I	0.7237	0.0592	4.77E-04	0.60764	0.83969	135.5	0.33333	0.66667	0.66667	32	12	16	24	0.6667	0.6667	0.7273	0.6000
Tsum	0.7156	0.06128	7.60E-04	0.59546	0.83567	331	0.36111	0.64583	0.63889	31	13	17	23	0.6458	0.6389	0.7045	0.5750
Nasal	0.6369	0.06423	0.03254	0.51097	0.76275	72.5	0.38889	0.64583	0.61111	31	14	17	22	0.6458	0.6111	0.6889	0.5641
Nasal S	0.6285	0.06457	0.04479	0.50192	0.75503	112.5	0.38889	0.60417	0.61111	29	14	19	22	0.6042	0.6111	0.6744	0.5366
Nasal I	0.673	0.06398	0.00688	0.54763	0.79844	103	0.36111	0.64583	0.63889	31	13	17	23	0.6458	0.6389	0.7045	0.5750
**Ratio 15deg**	State	8 missing data points															
positive	39																
negative	37																
	**Area**	**SE**	**Asymptotic F**	**95% LCL**	**95% UCL**	**cut off**	**1-SPEC**	**SEN**	**SPEC**	**a**	**b**	**c**	**d**	**Sens**	**Spfc**	**PPV**	**NPV**
RCG vol	0.6642	0.06473	0.01378	0.53738	0.7911	0.435	0.37838	0.64103	0.62162	25	14	14	23	0.6410	0.6216	0.6410	0.6216
gRNFL	0.6438	0.06725	0.03105	0.51198	0.77561	91.5	0.37838	0.61538	0.62162	24	14	15	23	0.6154	0.6216	0.6316	0.6053
tRNFL	0.7072	0.06125	0.00189	0.58717	0.82725	64.5	0.35135	0.66667	0.64865	26	13	13	24	0.6667	0.6486	0.6667	0.6486
Temp S	0.5956	0.06765	0.15153	0.46304	0.72823	128.5	0.40541	0.58974	0.59459	23	15	16	22	0.5897	0.5946	0.6053	0.5789
Temp I	0.6715	0.06555	0.01011	0.54304	0.8	133.5	0.32432	0.66667	0.67568	26	12	13	25	0.6667	0.6757	0.6842	0.6579
Tsum	0.6552	0.06747	0.01992	0.52299	0.78747	327.5	0.35135	0.66667	0.64865	26	13	13	24	0.6667	0.6486	0.6667	0.6486
Nasal	0.5797	0.06744	0.23204	0.44751	0.71188	71.5	0.45946	0.53846	0.54054	21	17	18	20	0.5385	0.5405	0.5526	0.5263
Nasal S	0.5939	0.06773	0.15908	0.46115	0.72665	111.5	0.45946	0.5641	0.54054	22	17	17	20	0.5641	0.5405	0.5641	0.5405
Nasal I	0.6608	0.06477	0.01591	0.53383	0.78772	99.5	0.37838	0.61538	0.62162	24	14	15	23	0.6154	0.6216	0.6316	0.6053

**RIGHT EYE**																	
**RE PVEP**	State																
positive	12									RE	RE	RE	RE				
negative	30									1	0	1	0	a/(a+c)	d/(b+d)	a/(a+b)	d/(c+d)
	**Area**	**SE**	**Asymptotic F**	**95% LCL**	**95% UCL**	**cut off**	**1-SPEC**	**SEN**	**SPEC**	**a**	**b**	**c**	**d**	**Sens**	**Spfc**	**PPV**	**NPV**
RCG vol	0.825	0.06621	0.00112	0.69524	0.95476	0.39	0.16667	0.83333	0.83333	10	5	2	25	0.8333	0.8333	0.6667	0.9259
gRNFL	0.7792	0.07834	0.00514	0.62562	0.93271	87.5	0.26667	0.75	0.73333	9	8	3	22	0.7500	0.7333	0.5294	0.8800
tRNFL	0.8375	0.06743	7.17E-04	0.70533	0.96967	53	0.16667	0.83333	0.83333	10	5	2	25	0.8333	0.8333	0.6667	0.9259
Temp S	0.7486	0.08484	0.01271	0.58233	0.91489	121.5	0.26667	0.75	0.73333	9	8	3	22	0.7500	0.7333	0.5294	0.8800
Temp I	0.7181	0.08562	0.02884	0.55024	0.88587	116.5	0.26667	0.75	0.73333	9	8	3	22	0.7500	0.7333	0.5294	0.8800
Tsum	0.7653	0.08075	0.00784	0.60701	0.92355	299	0.26667	0.75	0.73333	9	8	3	22	0.7500	0.7333	0.5294	0.8800
Nasal	0.7569	0.08471	0.01001	0.59092	0.92297	64	0.26667	0.75	0.73333	9	8	3	22	0.7500	0.7333	0.5294	0.8800
Nasal S	0.625	0.09729	0.21024	0.43431	0.81569	108.5	0.43333	0.58333	0.56667	7	13	5	17	0.5833	0.5667	0.3500	0.7727
Nasal I	0.7569	0.07956	0.01001	0.60101	0.91287	87.5	0.26667	0.75	0.73333	9	8	3	22	0.7500	0.7333	0.5294	0.8800
**RE slope**	State																
positive	7																
negative	35									1	0	1	0	a/(a+c)	d/(b+d)	a/(a+b)	d/(c+d)
	**Area**	**SE**	**Asymptotic F**	**95% LCL**	**95% UCL**	**cut off**	**1-SPEC**	**SEN**	**SPEC**	**a**	**b**	**c**	**d**	**Sens**	**Spfc**	**PPV**	**NPV**
RCG vol	0.6959	0.08653	0.10523	0.52633	0.86551	0.39	0.28571	0.71429	0.71429	5	10	2	25	0.7143	0.7143	0.3333	0.9259
gRNFL	0.6041	0.10253	0.38945	0.40312	0.80504	89.5	0.42857	0.57143	0.57143	4	15	3	20	0.5714	0.5714	0.2105	0.8696
tRNFL	0.6612	0.11912	0.18249	0.42775	0.8947	53	0.28571	0.71429	0.71429	5	10	2	25	0.7143	0.7143	0.3333	0.9259
Temp S	0.6469	0.10679	0.22437	0.43764	0.85624	126	0.42857	0.57143	0.57143	4	15	3	20	0.5714	0.5714	0.2105	0.8696
Temp I	0.5286	0.11502	0.81324	0.30314	0.754	121.5	0.42857	0.57143	0.57143	4	15	3	20	0.5714	0.5714	0.2105	0.8696
Tsum	0.6061	0.10716	0.38022	0.39609	0.81616	313	0.42857	0.57143	0.57143	4	15	3	20	0.5714	0.5714	0.2105	0.8696
Nasal	0.6449	0.11341	0.23087	0.42262	0.86717	64	0.34286	0.71429	0.65714	5	12	2	23	0.7143	0.6571	0.2941	0.9200
Nasal S	0.5429	0.11854	0.72306	0.31052	0.77519	107.5	0.42857	0.57143	0.57143	4	15	3	20	0.5714	0.5714	0.2105	0.8696
Nasal I	0.5857	0.10097	0.47848	0.38781	0.78361	92	0.42857	0.57143	0.57143	4	15	3	20	0.5714	0.5714	0.2105	0.8696
**RE Ratio30deg**	State																
positive	26																
negative	16									1	0	1	0	a/(a+c)	d/(b+d)	a/(a+b)	d/(c+d)
	**Area**	**SE**	**Asymptotic F**	**95% LCL**	**95% UCL**	**cut off**	**1-SPEC**	**SEN**	**SPEC**	**a**	**b**	**c**	**d**	**Sens**	**Spfc**	**PPV**	**NPV**
RCG vol	0.7885	0.08377	0.00188	0.62427	0.95266	0.435	0.1875	0.80769	0.8125	21	3	5	13	0.8077	0.8125	0.8750	0.7222
gRNFL	0.7548	0.08517	0.00604	0.58788	0.92174	96.5	0.25	0.76923	0.75	20	4	6	12	0.7692	0.7500	0.8333	0.6667
tRNFL	0.7716	0.07358	0.00343	0.62741	0.91585	64	0.3125	0.65385	0.6875	17	5	9	11	0.6538	0.6875	0.7727	0.5500
Temp S	0.7789	0.07664	0.00266	0.62863	0.92906	133.5	0.3125	0.69231	0.6875	18	5	8	11	0.6923	0.6875	0.7826	0.5789
Temp I	0.7476	0.08266	0.00764	0.58559	0.9096	131.5	0.375	0.65385	0.625	17	6	9	10	0.6538	0.6250	0.7391	0.5263
Tsum	0.7764	0.07891	0.0029	0.62178	0.9311	331	0.3125	0.69231	0.6875	18	5	8	11	0.6923	0.6875	0.7826	0.5789
Nasal	0.6827	0.0946	0.04902	0.49727	0.86811	74	0.3125	0.65385	0.6875	17	5	9	11	0.6538	0.6875	0.7727	0.5500
Nasal S	0.7512	0.08504	0.0068	0.58452	0.91788	113.5	0.25	0.76923	0.75	20	4	6	12	0.7692	0.7500	0.8333	0.6667
Nasal I	0.6911	0.09335	0.03948	0.50814	0.87407	97.5	0.3125	0.69231	0.6875	18	5	8	11	0.6923	0.6875	0.7826	0.5789
**RE Ratio15deg**	State																
positive	23																
negative	15									1	0	1	0	a/(a+c)	d/(b+d)	a/(a+b)	d/(c+d)
	**Area**	**SE**	**Asymptotic F**	**95% LCL**	**95% UCL**	**cut off**	**1-SPEC**	**SEN**	**SPEC**	**a**	**b**	**c**	**d**	**Sens**	**Spfc**	**PPV**	**NPV**
RCG vol	0.5565	0.10498	0.56033	0.35076	0.76229	0.425	0.46667	0.47826	0.53333	11	7	12	8	0.4783	0.5333	0.6111	0.4000
gRNFL	0.5928	0.10669	0.33925	0.38365	0.80186	94	0.33333	0.65217	0.66667	15	5	8	10	0.6522	0.6667	0.7500	0.5556
tRNFL	0.6464	0.0993	0.13152	0.45176	0.841	64	0.26667	0.65217	0.73333	15	4	8	11	0.6522	0.7333	0.7895	0.5789
Temp S	0.6058	0.10545	0.2757	0.39912	0.81247	131.5	0.4	0.6087	0.6	14	6	9	9	0.6087	0.6000	0.7000	0.5000
Temp I	0.5884	0.10586	0.36237	0.38092	0.79589	126	0.4	0.56522	0.6	13	6	10	9	0.5652	0.6000	0.6842	0.4737
Tsum	0.6174	0.10819	0.22647	0.40533	0.82945	331	0.33333	0.69565	0.66667	16	5	7	10	0.6957	0.6667	0.7619	0.5882
Nasal	0.5565	0.10159	0.56033	0.3574	0.75564	73.5	0.4	0.56522	0.6	13	6	10	9	0.5652	0.6000	0.6842	0.4737
Nasal S	0.5261	0.10633	0.7881	0.31769	0.73448	108.5	0.46667	0.52174	0.53333	12	7	11	8	0.5217	0.5333	0.6316	0.4211
Nasal I	0.613	0.10228	0.24414	0.41258	0.81351	95	0.4	0.6087	0.6	14	6	9	9	0.6087	0.6000	0.7000	0.5000

**LEFT EYE**																	
**LE PVEP**	State																
positive	13									LE	LE	LE	LE				
negative	29									1	0	1	0	a/(a+c)	d/(b+d)	a/(a+b)	d/(c+d)
	**Area**	**SE**	**Asymptotic F**	**95% LCL**	**95% UCL**	**cut off**	**1-SPEC**	**SEN**	**SPEC**	**a**	**b**	**c**	**d**	**Sens**	**Spfc**	**PPV**	**NPV**
RCG vol	0.9125	0.0516	2.32926E-05	0.81134	1.01359	0.39	0.06897	0.92308	0.93103	12	2	1	27	0.92308	0.931034	0.8571429	0.9642857
gRNFL	0.8899	0.06512	0.000063483	0.7623	1.01754	84	0.10345	0.92308	0.89655	12	3	1	26	0.92308	0.896552	0.8	0.962963
tRNFL	0.9111	0.05217	2.47427E-05	0.80888	1.0134	51.5	0.06897	0.92308	0.93103	12	2	1	27	0.92308	0.931034	0.8571429	0.9642857
Temp S	0.7971	0.07632	0.00231	0.6475	0.94666	119	0.27586	0.69231	0.72414	9	8	4	21	0.69231	0.724138	0.5294118	0.84
Temp I	0.8873	0.06483	7.11915E-05	0.7602	1.01434	103.5	0.06897	0.84615	0.93103	11	2	2	27	0.84615	0.931034	0.8461538	0.9310345
Tsum	0.8886	0.06524	6.72328E-05	0.76072	1.01647	277.5	0.10345	0.92308	0.89655	12	3	1	26	0.92308	0.896552	0.8	0.962963
Nasal	0.7546	0.0814	0.009	0.59511	0.91418	62.5	0.31034	0.69231	0.68966	9	9	4	20	0.69231	0.689655	0.5	0.8333333
Nasal S	0.752	0.08012	0.00975	0.59495	0.90903	112.5	0.37931	0.61538	0.62069	8	11	5	18	0.61538	0.62069	0.4210526	0.7826087
Nasal I	0.8501	0.06851	0.000328949	0.71585	0.98442	91	0.17241	0.84615	0.82759	11	5	2	24	0.84615	0.827586	0.6875	0.9230769
**LE N95 slope**	State																
positive	7																
negative	35									1	0	1	0	a/(a+c)	d/(b+d)	a/(a+b)	d/(c+d)
	**Area**	**SE**	**Asymptotic F**	**95% LCL**	**95% UCL**	**cut off**	**1-SPEC**	**SEN**	**SPEC**	**a**	**b**	**c**	**d**	**Sens**	**Spfc**	**PPV**	**NPV**
RCG vol	0.9388	0.05486	0.000285491	0.83125	1.0463	0.29	0.14286	0.85714	0.85714	6	5	1	30	0.85714	0.857143	0.5454545	0.9677419
gRNFL	0.9286	0.06693	0.000394484	0.79739	1.05975	75	0.14286	0.85714	0.85714	6	5	1	30	0.85714	0.857143	0.5454545	0.9677419
tRNFL	0.8755	0.10191	0.0019	0.67578	1.07524	44.5	0.2	0.85714	0.8	6	7	1	28	0.85714	0.8	0.4615385	0.9655172
Temp S	0.9163	0.06074	0.000576371	0.79728	1.03537	113.5	0.2	0.85714	0.8	6	7	1	28	0.85714	0.8	0.4615385	0.9655172
Temp I	0.9204	0.07852	0.000508484	0.76652	1.0743	97.5	0.14286	0.85714	0.85714	6	5	1	30	0.85714	0.857143	0.5454545	0.9677419
Tsum	0.9306	0.0735	0.000369978	0.78655	1.07467	250.5	0.14286	0.85714	0.85714	6	5	1	30	0.85714	0.857143	0.5454545	0.9677419
Nasal	0.7735	0.10433	0.02374	0.56899	0.97795	64.5	0.42857	0.57143	0.57143	4	15	3	20	0.57143	0.571429	0.2105263	0.8695652
Nasal S	0.8592	0.08431	0.00298	0.69393	1.02444	102	0.28571	0.71429	0.71429	5	10	2	25	0.71429	0.714286	0.3333333	0.9259259
Nasal I	0.9122	0.07269	0.000652621	0.76978	1.05471	83.5	0.17143	0.85714	0.82857	6	6	1	29	0.85714	0.828571	0.5	0.9666667
**LE Ratio30deg**	State																
positive	22																
negative	20									1	0	1	0	a/(a+c)	d/(b+d)	a/(a+b)	d/(c+d)
	**Area**	**SE**	**Asymptotic F**	**95% LCL**	**95% UCL**	**cut off**	**1-SPEC**	**SEN**	**SPEC**	**a**	**b**	**c**	**d**	**Sens**	**Spfc**	**PPV**	**NPV**
RCG vol	0.6625	0.09517	0.07175	0.47597	0.84903	0.4	0.1	0.59091	0.9	13	2	9	18	0.59091	0.9	0.8666667	0.6666667
gRNFL	0.6432	0.09563	0.1126	0.45575	0.83061	92.5	0.4	0.59091	0.6	13	8	9	12	0.59091	0.6	0.6190476	0.5714286
tRNFL	0.6909	0.08754	0.03439	0.51934	0.86248	64.5	0.45	0.59091	0.55	13	9	9	11	0.59091	0.55	0.5909091	0.55
Temp S	0.5761	0.09712	0.39885	0.38578	0.76649	126	0.45	0.54545	0.55	12	9	10	11	0.54545	0.55	0.5714286	0.5238095
Temp I	0.6955	0.08573	0.03032	0.52744	0.86347	138.5	0.3	0.68182	0.7	15	6	7	14	0.68182	0.7	0.7142857	0.6666667
Tsum	0.6557	0.09284	0.0845	0.47372	0.83764	331	0.4	0.59091	0.6	13	8	9	12	0.59091	0.6	0.6190476	0.5714286
Nasal	0.5932	0.09051	0.30181	0.41579	0.77057	70.5	0.45	0.54545	0.55	12	9	10	11	0.54545	0.55	0.5714286	0.5238095
Nasal S	0.5216	0.09554	0.81091	0.33435	0.70884	116.5	0.55	0.45455	0.45	10	11	12	9	0.45455	0.45	0.4761905	0.4285714
Nasal I	0.6455	0.09013	0.10701	0.4688	0.8221	105.5	0.4	0.59091	0.6	13	8	9	12	0.59091	0.6	0.6190476	0.5714286
**LE Ratio15deg**	State																
positive	16																
negative	22									1	0	1	0	a/(a+c)	d/(b+d)	a/(a+b)	d/(c+d)
	**Area**	**SE**	**Asymptotic F**	**95% LCL**	**95% UCL**	**cut off**	**1-SPEC**	**SEN**	**SPEC**	**a**	**b**	**c**	**d**	**Sens**	**Spfc**	**PPV**	**NPV**
RCG vol	0.7656	0.07849	0.0057	0.6118	0.91945	0.43	0.31818	0.6875	0.68182	11	7	5	15	0.6875	0.681818	0.6111111	0.75
gRNFL	0.7031	0.08702	0.03452	0.53257	0.87368	92.5	0.40909	0.625	0.59091	10	9	6	13	0.625	0.590909	0.5263158	0.6842105
tRNFL	0.7699	0.07683	0.00497	0.6193	0.92047	63.5	0.27273	0.6875	0.72727	11	6	5	16	0.6875	0.727273	0.6470588	0.7619048
Temp S	0.5994	0.09288	0.30076	0.41739	0.78147	126	0.45455	0.5625	0.54545	9	10	7	12	0.5625	0.545455	0.4736842	0.6315789
Temp I	0.7457	0.08174	0.01054	0.58552	0.90596	135.5	0.27273	0.75	0.72727	12	6	4	16	0.75	0.727273	0.6666667	0.8
Tsum	0.696	0.08753	0.04135	0.52446	0.86759	327	0.36364	0.625	0.63636	10	8	6	14	0.625	0.636364	0.5555556	0.7
Nasal	0.6293	0.09126	0.17855	0.45039	0.80813	68	0.45455	0.5625	0.54545	9	10	7	12	0.5625	0.545455	0.4736842	0.6315789
Nasal S	0.6534	0.08952	0.11037	0.47796	0.82886	114	0.45455	0.5625	0.54545	9	10	7	12	0.5625	0.545455	0.4736842	0.6315789
Nasal I	0.7003	0.08615	0.03713	0.53143	0.86913	104.5	0.31818	0.6875	0.68182	11	7	5	15	0.6875	0.681818	0.6111111	0.75
